# The Impaired Bioenergetics of Diabetic Cardiac Microvascular Endothelial Cells

**DOI:** 10.3389/fendo.2021.642857

**Published:** 2021-05-14

**Authors:** Haitao Zhang, Yan Shen, Il-man Kim, Neal L. Weintraub, Yaoliang Tang

**Affiliations:** ^1^ Vascular Biology Center, Medical College of Georgia, Augusta University, Augusta, GA, United States; ^2^ Anatomy, Cell Biology & Physiology, School of Medicine, Indiana University, Indianapolis, IN, United States

**Keywords:** diabetes, fatty acid oxidation, mitochondrial oxidative metabolism, glycolysis, cardiac microvascular, endothelial cells

## Abstract

Diabetes causes hyperglycemia, which can create a stressful environment for cardiac microvascular endothelial cells (CMECs). To investigate the impact of diabetes on the cellular metabolism of CMECs, we assessed glycolysis by quantifying the extracellular acidification rate (ECAR), and mitochondrial oxidative phosphorylation (OXPHOS) by measuring cellular oxygen consumption rate (OCR), in isolated CMECs from wild-type (WT) hearts and diabetic hearts (db/db) using an extracellular flux analyzer. Diabetic CMECs exhibited a higher level of intracellular reactive oxygen species (ROS), and significantly reduced glycolytic reserve and non-glycolytic acidification, as compared to WT CMECs. In addition, OCR assay showed that diabetic CMECs had increased maximal respiration, and significantly reduced non-mitochondrial oxygen consumption and proton leak. Quantitative PCR (qPCR) showed no difference in copy number of mitochondrial DNA (mtDNA) between diabetic and WT CMECs. In addition, gene expression profiling analysis showed an overall decrease in the expression of essential genes related to β-oxidation (Sirt1, Acox1, Acox3, Hadha, and Hadhb), tricarboxylic acid cycle (TCA) (Idh-3a and Ogdh), and electron transport chain (ETC) (Sdhd and Uqcrq) in diabetic CMECs compared to WT CMECs. Western blot confirmed that the protein expression of Hadha, Acox1, and Uqcrq was decreased in diabetic CMECs. Although lectin staining demonstrated no significant difference in capillary density between the hearts of WT mice and db/db mice, diabetic CMECs showed a lower percentage of cell proliferation by Ki67 staining, and a higher percentage of cellular apoptosis by TUNEL staining, compared with WT CMECs. In conclusion, excessive ROS caused by hyperglycemia is associated with impaired glycolysis and mitochondrial function in diabetic CMECs, which in turn may reduce proliferation and promote CMEC apoptosis.

## Introduction

Obesity is the leading cause of insulin resistance and drives obesity-associated type 2 diabetes, which is highly prevalent in Western countries (1, 2). Diabetes is a major cause of morbidity and mortality in the Western world (3). Over time, impaired function of pancreatic islets in type 2 diabetes patients lead to persistent hyperglycemia, which is associated with diabetic micro-vascular and macro-vascular complications, including acute myocardial infarction, diabetic retinopathy, cerebrovascular accident, and lower-limb amputation (4, 5).

Diabetic patients have elevated plasma levels of lipoproteins and triglycerides, which can cause vascular endothelial damage (6, 6). Additionally, sustained hyperglycemia associated with increased oxidative stress impairs endothelial cell replication and accelerates endothelial cell apoptosis *via* mitochondrial dysfunction in both type 1 and type 2 diabetes (7–9). Both oxidative phosphorylation (OXPHOS) and glycolysis are functionally important for angiogenic responses of vascular endothelial cells (10). Although OXPHOS results in higher ATP production, endothelial cells rely heavily on glycolysis to rapidly meet their energy and metabolic demands (11, 12).

Type 2 diabetes mellitus is characterized by abnormalities in insulin signaling, fatty acid metabolism, and mitochondrial OXPHOS (13). Adenosine triphosphate (ATP) is generated in mitochondria *via* OXPHOS using glucose metabolism-derived acetyl-CoA and fatty acid metabolism-derived acyl-CoA as energy substrates (14). In general, type 2 diabetes is associated with increased fatty acid utilization and reduced glucolytic metabolism. Notably, pathways that allow endothelial cells to upregulate glycolysis in response to hypoxia are impaired in diabetes (15). Dodd et al. (16) reported that reduced succinate content leads to decreased hypoxia-inducible factor 1-α (HIF1-α) signaling in diabetic hearts. Moreover, hyperglycemia-induced oxidative stress depletes nicotinamide adenine dinucleotide (NAD+), impairs glycolysis, and inhibits electron transport and ATP generation, therefore promoting endothelial dysfunction and diabetic vascular complications (17), suggesting that diabetes-associated oxidative stress might also impair pathways that allow endothelial cells to upregulate OXPHOS.

The impact of diabetes on fatty acid metabolism and glycolysis in cardiac microvascular endothelial cells (CMECs) is not well studied. To study the cellular metabolism of diabetic CMECs, we assessed glycolysis by quantifying the extracellular acidification rate (ECAR), and OXPHOS by measuring cellular oxygen consumption rate (OCR), in isolated CMECs from wide-type C57BL/6 mouse hearts (WT) and diabetic mouse hearts (db/db). We found that diabetic CMECs showed decreased glycolytic reserve but significantly increased maximal respiration, with no change in the copy number of mitochondrial DNA compared with WT CMECs. Furthermore, gene expression profiling studies in diabetic CMECs versus WT CMECs revealed an overall inhibition of the expression of essential genes related to β-oxidation, tricarboxylic acid cycle (TCA), and electron transport chain (ETC).

## Materials and Methods

### CMECs Isolation and Culture

Animals were treated according to approved protocols and animal welfare regulations of the Institutional Animal Care and Use Committee of the Medical College of Georgia, Augusta University. Cardiac microvascular endothelial cells (CMECs) were isolated from the hearts of male C57BL/6 (WT) and diabetic (db/db) mice (The Jackson Laboratory, Bar Harbor, ME, USA) using the enzyme dissociation method. Briefly, mice were euthanized, and ventricular cardiac tissues were minced into 1 mm^3^ size and digested with 0.1% collagenase IV and 1 U/mL dispase in DMEM medium. Then, the cells were subjected to CD31 positive magnetic-activated cell sorting (MACS) using CD31 microBeads in combination with MS columns (Miltenyi Biotec Inc, Auburn, CA). The isolated CD31-positive cells were plated on fibronectin/gelatin coated wells (0.5 mg fibronectin in 100 ml 0.1% gelatin) and cultured in endothelial cell medium (Cell Biologics, Chicago, IL) containing 5% fetal bovine serum, VEGF, heparin, EGF, ECGS, hydrocortisone, L-glutamine and antibiotic-antimycotic.

### Body Weight and Blood Glucose Measurement

For measuring non-fasting blood glucose, the blood glucose was measured directly on a drop of blood from the mouse tail by a disposable test trip using EvenCare^G2^ glucometer (Medline, Taiwan).

### Seahorse Analysis of Mitochondrial Respirometry

OCR and ECAR were quantified using Seahorse XF 96 extracellular flux analyzer (Agilent Technologies, Lexington, MA, USA) as previously described with minor modifications (18, 19). Briefly, the cells were plated at 10,000 cells/well in the Seahorse XF cell culture microplate. The sensor cartridge for XF analyzer was hydrated in a 37°C non-CO_2_ incubator the day prior to experimentation. On the day of the Seahorse assay, the medium was changed to Seahorse XF DMEM base medium without phenol red. For OCR assay, the medium was supplemented with 10 mM glucose, 2 mM L-glutamine, and 1 mM pyruvate, pH 7.4, and metabolic parameters were measured using the Mitochondrial Stress Test Kit (Agilent, Cat. No. 103015-100, Cedar Creek, TX). OCR assay was performed using the following protocol after calibration and equilibration: four cycles of wait 3 min, mix 3 min, measure 3 min (basal value) and then measure values after each of the following injections (see **Figure 2A** for a description): 2.5 µM oligomycin (final concentration), 2 µM carbonyl cyanide-p-trifluoromethoxyphenyl-hydrazon (FCCP) (final concentration), and 0.5 µM rotenone/antimycin A (Rot/AA) (final concentration). For ECAR assay, the medium was supplemented with 2 mM L-glutamine, and 1 mM pyruvate, pH 7.4, and metabolic parameters were measured using the Glycolytic Rate Assay kit (Agilent, Cat. No. 103344-100, Cedar Creek, TX). ECAR assay was performed using the following protocol after calibration and equilibration: four cycles of wait 3 min, mix 3 min, measure 3 min (basal value) and then measure values after each of the following injections (see **Figure 3A** for a description): 10 mM glucose (final concentration), 0.5 µM rotenone/antimycin A (final concentration), and 50 mM 2-Deoxy-D-glucose (2-DG) (final concentration). Post assay, wells were washed, cells lysed, and a bicinchoninic acid assay (BCA) protein assay was performed for protein content. Data presented are normalized for total protein per well. Each point represents an average of 8 to 12 different wells.

### Isolation and Quantification of Genomic DNA and Messenger RNA

Genomic DNA was extracted from CMECs with the QIAamp DNA blood mini kit (QIAGEN, Valencia, CA) following the manufacturer’s instructions.

Total RNA was extracted by RNAzol RT (Molecular Research Center, Inc, Cincinnati, OH, USA) following the manufacturer’s instructions. cDNA was synthesized from total RNA using the RevertAid First Strand cDNA Synthesis kit (Thermo Scientific). The cDNA was used to perform quantitative PCR on a CFX96 Touch Real-Time PCR Detection System (Bio-Rad Laboratories, Hercules, CA) using PowerUp SYBR Green Master Mix (Thermo Fisher, Waltham, MA). Amplification was performed at 50°C for 2 min, 95°C for 2 min, followed by 45 cycles of 95°C for 15 s, 55°C for 15s, and 72°C for 1 min. The primer sequences are listed in **Table 1**.

**Table 1:** Primer list.

Gene	Sequence (5′–3′)
*β-actin FWD* (mouse)	AGAGCATAGCCCTCGTAGAT
*β-actin REV* (mouse)	GCTGTGCTGTCCCTGTATG
*Sirt1 FWD* (mouse)	GTTGGTGGCAACTCTGATAAATG
*Sirt1 REV* (mouse)	GTCATAGGCTAGGTGGTGAATATG
*Acox1 FWD* (mouse)	CCTTGGCCAATGCTCTCATTA
*Acox1 REV* (mouse)	CGCAGCAGTATAAACTCTTCCC
*Acox3 FWD* (mouse)	CCCTAGAGAAGCTACGAGAACT
*Acox3 REV* (mouse)	CAGGCAGTTAATCAGCACTAGAA
*Hadha FWD* (mouse)	CCATGTCGGCCTTCTCAAA
*Hadha REV* (mouse)	AGTGAAGAAGAAAGCTCTCACAT
*Hadhb FWD* (mouse)	AGACCATGGGCCACTCT
*Hadhb REV* (mouse)	CTTCTTGGCCAGACTATGAGAAC
*Idh3a FWD* (mouse)	GGCCATCCATCTATGAATCTGT
*Idh3a REV* (mouse)	GTATTCTCCTTCCGTGTTCTCTC
*Ogdh FWD* (mouse)	CATGTATCACCGCAGGATCAA
*Ogdh REV* (mouse)	GGTCTTTCCCATCACGACAG
*Sdhd FWD* (mouse)	GATGCCGACATCGTGGTAAT
*Sdhd REV* (mouse)	GTTACCGACTACGTTCATGGG
*Uqcrq FWD* (mouse)	CTTTGCTGAAATAGCTTGGGAAG
*Uqcrq REV* (mouse)	GAACCTGGCGCGGATAC
*MT-ND6 FWD* (mouse)	CACCCAGCTACTACCATCATTC
*MT-ND6 REV* (mouse)	GTTTGGGAGATTGGTTGATGTATG
*POLB FWD* (mouse)	GGCGGATGGTGTACTCATT
*POLB REV* (mouse)	ACTGTGGTGTTCTCTACTTCAC

### Western Blotting Assay

Western blotting was performed as previously described (18, 19). Briefly, the protein samples were separated on 10% SDS–polyacrylamide gels, and the gel was then transferred onto nitrocellulose membranes (LI-COR Biosciences). The membranes were blocked with Intercept™ blocking buffer (LI-COR Biosciences) and blotted with rabbit anti-HADHA (1:1000; 10758-1-ap, Proteintech), rabbit anti-ACOX1 (1:1000; 10957-1-ap, Proteintech), rabbit anti-UQCRQ (1:1000; 14975-1-ap, Proteintech), and mouse anti-GAPDH (1:5000, MAB374, EMD Millipore) overnight at 4°C. After washing with 1 × TBST, the membranes were tagged with IRDye 680 goat anti-rabbit IgG and IRDye 800 goat anti-mouse IgG (1:10,000, LI-COR Biosciences) at room temperature for 1 h. The probed blot was scanned using an Odyssey infrared imaging system (LI-COR Biosciences).

### Total ROS Detection in Endothelial Cells

ROS was measured using the total ROS-ID Total ROS Detection Kit (ENZ-51011, Enzo Life Sciences, Farmingdale, NY) according to the manufacturer’s instructions. Briefly, CMEC from WT and db/db mouse hearts were seeded in 35mm glass-bottom culture dishes (P35G-1.5-10-C, MatTek, Ashland, MA) overnight. ROS in the cells were detected by staining with the Oxidative Stress Detection Reagent (Green). NucBlue^®^ Live Cell Stain ReadyProbe™ reagent (R37605, Thermo) was used to counterstain cells.

### Histological Analyses

To quantify capillary density in hearts, 100µl Griffonia Simplicifolia lectin at 1mg/ml (L-1100, Vector Laboratories, Burlingame, CA) was injected into the right jugular vein. The hearts were fixed with 10% formalin followed by 30% sucrose, frozen in OCT and processed for sectioning. Heart slides were blocked with 10% donkey serum, and then stained overnight at 4°C with goat Anti-Griffonia Simplicifolia Lectin (1:200, Vector laboratories). Slides were incubated with donkey anti-goat secondary antibody conjugated to Alexa 555 (1:400, Life Technologies, Carlsbad, CA); Slides were mounted using VECTASHIELD HardSet mount media with DAPI. Staining was visualized using a Zeiss 780 laser scanning microscope (Carl Zeiss, Thornwood, NY). Capillary density was calculated as the number of capillaries per mm^2^.

To quantify cell proliferation in cardiac endothelial cells, we performed double immunostaining with Ki-67 for the analysis of cell proliferation, and CD31 for microvessels. After carrying out epitope retrieval using citrate buffer in a 2100 Retriever (Prestige Medical, Model No. 210050, England), we blocked tissue section with 5% goat serum and streptavidin/biotin (Vector laboratories, Inc. Burlingame, CA). Heart sections were stained overnight at 4°C with biotinylated anti-mouse/rat Ki67 (1:100, eBioscience) and rabbit anti-CD31 (1:100, Cell Signaling Technology). Slides were incubated with streptavidin Alexa Fluor 488 conjugate (1:400, Life Technologies, Carlsbad, CA) and Alexa Fluor555 conjugated goat anti-rabbit IgG (1:400, Life Technologies, Carlsbad, CA). Cell proliferation was calculated by the percentage of Ki67 positive nuclei to the total number of CD31 positive cells in each field.

Cell apoptosis was assessed by dual immunofluorescence staining for CD31 and terminal deoxynucleotidyl-transferase dUTP nick-end-labeling (TUNEL) in heart tissues as we described previously (19). After epitope retrieval, heart sections were stained overnight at 4°C with rabbit anti-CD31 (1:100, Cell Signaling Technology), followed by TUNEL staining next day using DeadEnd™ Fluorometric TUNEL System (Promega, Madison, WI). Slides were then incubated with Alexa Fluor555 conjugated goat anti-rabbit IgG (1:400, Life Technologies, Carlsbad, CA). Slides were mounted using VECTASHIELD HardSet Mounting Medium with DAPI (Vector Laboratories), cell apoptosis was classified as a percentage of TUNEL positive nuclei in relation to the total number of CD31 positive cells in each field.

### Statistical Analysis

All data were expressed as the mean± standard error of the mean (SEM). A two-tailed student’s t-test (GraphPad Prism version 7.0) was used to compare data between two groups. A value of P<0.05 was considered statistically significant.

## Results

### Diabetes Is Associated With Weight Gain and Hyperglycemia in Mice

**Figure 1:**
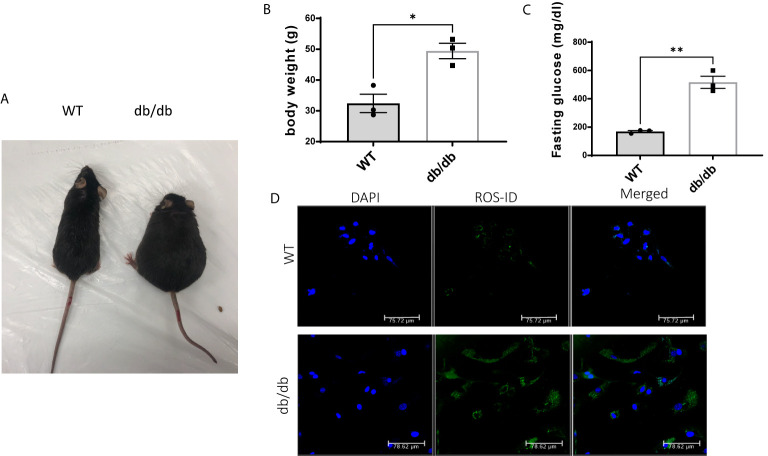
Comparison of WT and db/db mice. **(A)** A db/db mouse in comparison to an age-matched C57BL/6 WT mouse; **(B)** Body weight (g) in 6-month-old WT mice and db/db mice; results are shown as mean ± SEM (n = 3, * p < 0.05); **(C)** Blood glucose levels (mg/dl) in 6-month-old WT mice and db/db mice fed with standard chow, (n = 3, **p < 0.01); **(D)** Green fluorescence intensity was used as a measure of relative intracellular ROS in WT CMECs and diabetic CMECs.

As expected, db/db mice gained significantly more body weight (**Figures 1A, B**) and developed severe hyperglycemia compared to WT mice at 6 months of age (**Figure 1C**). Previous studies demonstrated that hyperglycemia associated with diabetes increases cellular reactive oxygen species (ROS) production and decreases cellular antioxidant defense capacity (20, 21). Accordingly, we detected increased intracellular ROS levels in CMECs from db/db hearts compared to WT hearts shown in **Figure 1D**.

### Diabetic CMECs Have Increased OXPHOS Maximal Respiratory Capacity

**Figure 2:**
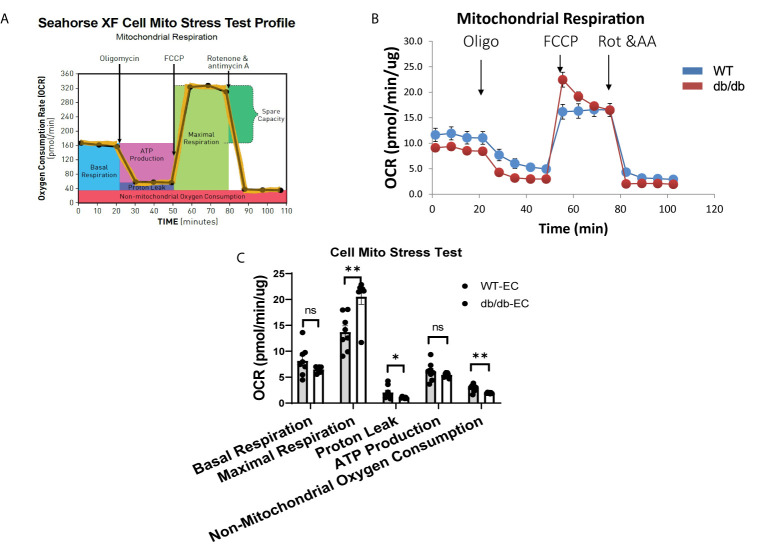
Assessment of oxygen consumption rate (OCR) in WT and diabetic CMECs. **(A)** Schematic representation of the protocol employed in data collection and calculations of mitochondrial respiration; **(B)** Normalized OCR data; **(C)** Parameters of mitochondrial respiration function calculated from the OCR tracing in **(B)**. Results are normalized to total cellular protein and shown as mean ± SEM (n = 8), NS p > 0.05, *p < 0.05, **p < 0.01.

To determine the impact of diabetes on CMEC metabolism, we monitored real-time cellular OCR following sequential addition of oligomycin, FCCP, rotenone, and antimycin A (**Figures 2A, B**). The results indicate that diabetic CMECs have significantly increased maximal respiration in comparison with WT CMECs (**Figure 2C**). Meanwhile, the proton leak and non-mitochondrial oxygen consumption were significantly decreased in diabetic CMECs compared to WT CMECs (**Figure 2C**). However, no significant change was noted for basal respiration or ATP production between diabetic CMECs and WT CMECs (**Figure 2C**).

### Diabetic CMECs Have Impaired Glycolytic Capacity

**Figure 3:**
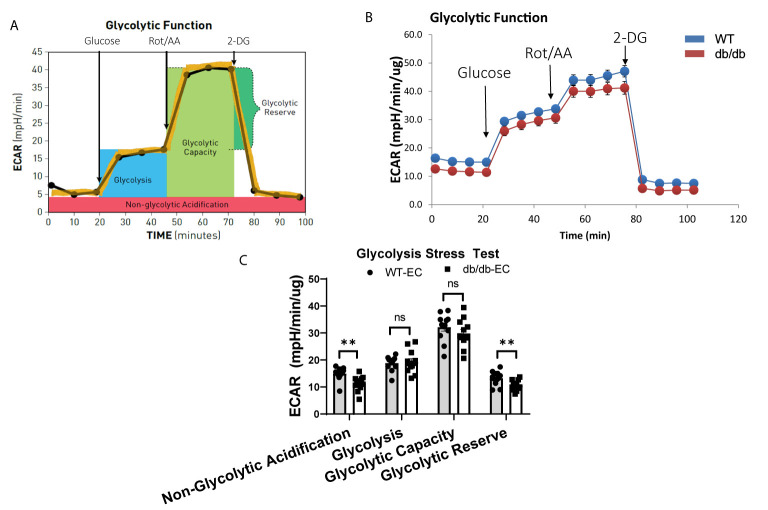
Assessment of extracellular acidification rate (ECAR) in WT and diabetic CMECs. **(A)** Schematic representation of the protocol employed in data collection and calculations of glycolytic function. **(B)** Normalized ECAR data. **(C)** Parameters of glycolytic function calculated from the ECAR tracing in **(B)**. Results are normalized to total cellular protein and shown as mean ± SEM (n = 12), ns p > 0.05, **p < 0.01.

To study the impact of diabetes on the glycolytic function of CMECs, we quantified ECAR, an index of cellular glycolytic capacity, in CMECs (**Figures 3A, B**). ECAR is comprised of both glycolytic and non-glycolytic acidification, and the non-glycolytic acidification was decreased in diabetic CMECs compared to WT CMECs (**Figure 3C**). Although no significant difference was detected in basal glycolysis or glycolytic capacity between diabetic CMECs and WT CMECs (**Figure 3C**), diabetic CMECs exhibited a lower capacity to increase the glycolytic rates, as reflected by the glycolytic reserve (**Figure 3C**).

### Diabetes Down-Regulates Genes Involved in Mitochondrial Fatty Acid β-Oxidation, Tricarboxylic Acid (TCA) Cycle, and Electron Transport Chain (ETC) in CMECs

**Figure 4:**
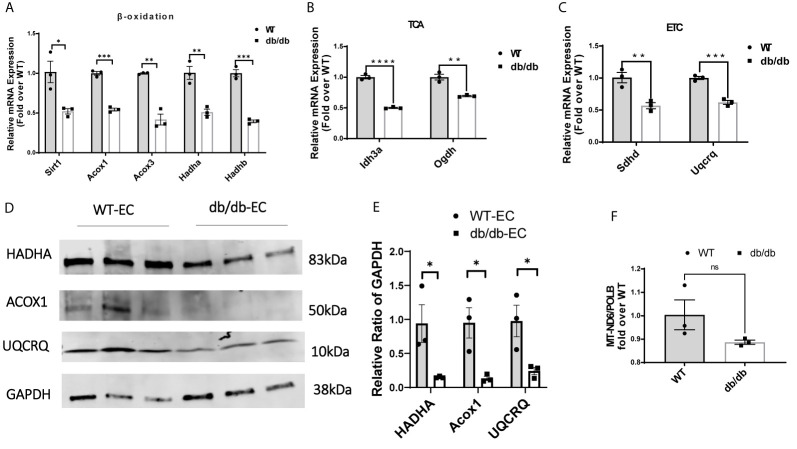
Comparison of relative mRNA levels of genes related to **(A)** fatty acid β-oxidation (Sirt1, Acox1, Acox3, Hadha and Hadhb), **(B)** TCA cycle (Idh3a and Ogdh), and **(C)** ETC (Sdhd and Uqcrq) in WT-CMECs and db/db-CMECs. The amount of mRNA was normalized using β-actin. TCA, tricarboxylic acid cycle; ETC, electron transport chain. Results are shown as mean ± SEM (n = 3), ns p > 0.05, *p < 0.05, **p < 0.01, ***p < 0.001, ****p < 0.0001. **(D, E)** Comparison of relative protein levels of enzymes related to fatty acid β-oxidation (Hadha and Acox1) and ETC (Uqcrq) in WT CMECs and diabetic CMECs. The amount of protein was normalized using GAPDH. Results are shown as mean ± SEM (n = 3), *P < 0.05. **(F)** Comparison of the relative copy number of mitochondria (MT)-encoded NADH-ubiquinone oxidoreductase chain 6 (MT-ND6) between WT CMECs and diabetic CMECs. The amount of mitochondrial DNA was normalized to the nuclear-encoded control gene DNA polymerase-β (PolB). Results are shown as mean ± SEM (n = 3), ns p > 0.05.

Next, we examined the transcription of a set of essential genes in fatty acid β-oxidation, TCA cycle, and ETC by quantitative real-time PCR (q-rtPCR). The results demonstrated that genes involved in fatty acid transport and oxidation (Sirt1, Acox1, Acox3, Hadha, Hadhb), TCA cycle (Idh3a and Ogdh), and ETC (Sdhd and Uqcrq) were significantly downregulated in diabetic CMECs compared to WT CMECs (**Figures 4A–C** **)**. We further examined the protein level of essential genes related to fatty acid oxidation and the ETC. As shown in **Figures 4D, E**, the expression of HADHA and Acox1 (related to fatty acid β-oxidation), and UQCRQ (component of the mitochondrial ETC), was significantly reduced in CMECs from diabetic mice in comparison to WT mice. The number of mitochondria can be estimated by quantifying the mitochondrial DNA copy number (22). To determine whether diabetes impacts the number of mitochondria in CMECs, we quantified the copy number of mitochondrial DNA (mtDNA) from genomic DNA isolated from CMECs. As shown in **Figure 4F**, the mitochondrial DNA copy numbers were similar in diabetic CMECs and WT CMECs.

### Effects of Diabetes on Cardiac Angiogenesis, Proliferation, and Apoptosis

**Figure 5:**
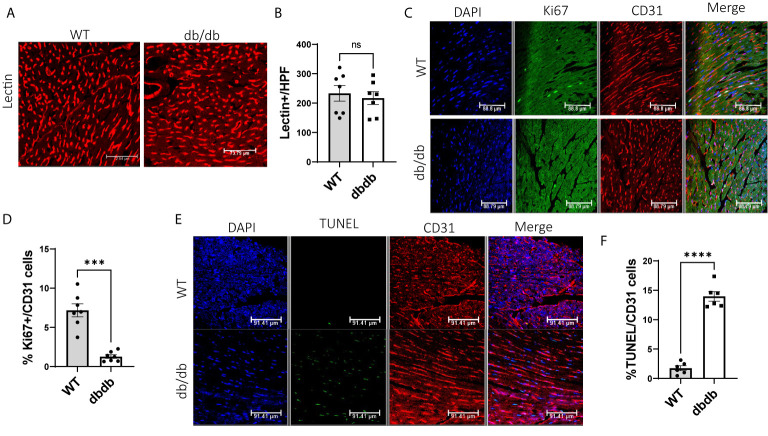
Comparison of cardiac angiogenesis, endothelial proliferation, and apoptosis between WT-hearts and db/db-hearts. **(A)** Immunofluorescent staining of lectin to detect capillary density in WT-hearts and db/db-hearts; **(B)** Comparison of Lectin-positive cells per high power field (HPF) between WT-hearts and db/db hearts (ns, P >0.05, n=7); **(C)** Immunofluorescent staining of Ki67 and CD31 to detect proliferative endothelial cells in WT-hearts and db/db-hearts; **(D)** The comparison of the percentage of Ki67^+^ endothelial cells between WT and db/db hearts (***P < 0.001, n=7); **(E)** Immunofluorescent staining of TUNEL and CD31 to detect apoptotic endothelial cells in WT-hearts and db/db hearts; **(F)** The comparison of the percentage of TUNEL-positive endothelial cells between WT and db/db hearts (****P < 0.0001, n=6).

To determine the impact of diabetes on cardiac angiogenesis, we measured the capillary density in heart tissues. Immunofluorescent staining for lectin showed no significant difference in capillary density between WT hearts and diabetic hearts (**Figures 5A, B**). However, we observed more proliferating cardiac endothelial cells in WT hearts than in diabetic hearts by dual-immunostaining for Ki67 and CD31 (**Figures 5C, D**), indicating that diabetes impairs proliferation of cardiac endothelial cells. Next, to study the impact of diabetes on apoptosis of cardiac endothelial cells, we performed immunofluorescent staining for TUNEL and CD31. We detected a significantly higher number of TUNEL+ apoptotic endothelial cells in diabetic hearts than in WT hearts, suggesting that diabetes increases apoptosis of cardiac endothelial cells (**Figures 5E, F**).

## Discussion

In this study, excessive ROS caused by hyperglycemia was associated with reduced glycolytic reserve, and reduced expression of enzymes involved in various metabolic pathways, in CMECs. Moreover, compared with WT CMECs, diabetic CMECs exhibited more cell apoptosis and less cell proliferation.

Angiogenesis is an energy-intensive process, since both OXPHOS and glycolysis are necessary for endothelial cell growth and angiogenic responses (10). Glucose is a primary energy source for normal endothelial cells, which are highly glycolytic. Repression of endothelial glycolysis by glucose transporter isoform1 (GLUT1) inhibition can impair developmental and pathological angiogenesis (23). In the hearts of diabetic patients, normal cardiac metabolism is dysregulated, as manifested as impaired glycolysis and greater dependence on fatty acid as a major energy source (24, 25). In diabetic patients, diabetes complications are caused in party by increased glucose influx into endothelial cells, which leads to impaired glycolysis and accumulation of glycolysis metabolites (such as methylglyoxal), and degradation of VEGFR2, leading to perturbations in angiogenesis (26, 27). Here, we observed that, compared with normal CMECs, the basal glycolysis rate in diabetic CMECs was not significantly reduced. However, the glycolytic reserve [the difference between glycolytic capacity and glycolysis (28)], was significantly reduced, indicating that the ability of diabetic CMECs to rely upon glycolysis to generate ATP in response to high metabolic demand is impaired.

According to reports, hyperglycemic stress can induce diabetic endothelial cells to transform from glycolysis to OXPHOS. Pyruvate kinase M (PKM) is a crucial enzyme that produces pyruvate, an essential oxidative fuel for metabolic transformation (12). However, a recent study showed that glucose oxidation in diabetic hearts is decreased, while rates of fatty acid oxidation (FAO) are increased, leading to oxidative stress damaging OXPHOS, and eventually causing mitochondrial dysfunction. In addition, mitochondrial dysfunction caused by metabolic stress leads to cardiomyocyte necrosis (29). In our real-time mitochondrial OCR assay, diabetic CMECs exhibited a significant increase in maximal respiration, which indicates that diabetic CMECs may preferentially convert their energy source from glycolysis to OXPHOS.

Our qRT-PCR data indicate that the diabetic CMECs have a general decrease in mRNA levels of OXPHOS-related genes, including fatty acid transport and oxidation (Sirt1, Acox1, Acox3, Hadha, Hadhb), TCA cycle(Idh3a, Ogdh), ETC (Uqcrq, Sdhd) compared with WT-CMECs. Western blots also confirmed decrease fatty acid β-oxidation and mitochondrial ETC enzyme protein levels. The down-regulation of OXPHOS-related genes might explain why diabetic CMECs exhibit decreased basal respiration and decreased ATP production, along with increased maximal respiration. The inhibition of OXPHOS-related gene expression could be caused by oxidative stress induced by hyperglycemia. Many studies have demonstrated that hyperglycemia increases ROS, contributing to diabetic endothelial apoptosis and dysfunction (30, 31). We observed increased cardiac endothelial cell apoptosis in db/db-hearts, a result consistent with the report by Peng C. et al. (25). They demonstrated that high glucose could induce apoptosis through increased ROS in cardiac microvascular endothelial cells. Oxidative stress has been shown to induce caspase-dependent apoptotic genes and inhibit metabolic gene expression (32, 33). These results are consistent with previous reports from other investigators. Li Q. et al. (34) reported that diabetes upregulates endothelial miR-34a by recruiting p66Shc through an oxidant-sensitive mechanism, thereby downregulating endothelial Sirt1 levels. ACOX1 and peroxisome proliferator-activated receptor-δ (PPAR-δ) were also significantly decreased in the muscle of diabetic rats (35). Recent studies have shown significantly reduced activity of HADHA, along with lipid accumulation, in diabetic hearts (36). Sdhd is one of the genes encoding mitochondrial complex I and II subunits and was found to be down-regulated in obese subjects and patients with type 2 diabetes (37).

For immuofluorescent staining of mouse CD31, a heat epitope retrieval process in citrate buffer is necessary. Interestingly, we observed that heat-induced epitope retrieval can increase the detection of nuclear antigens, such as Ki67.

**Figure 6:**
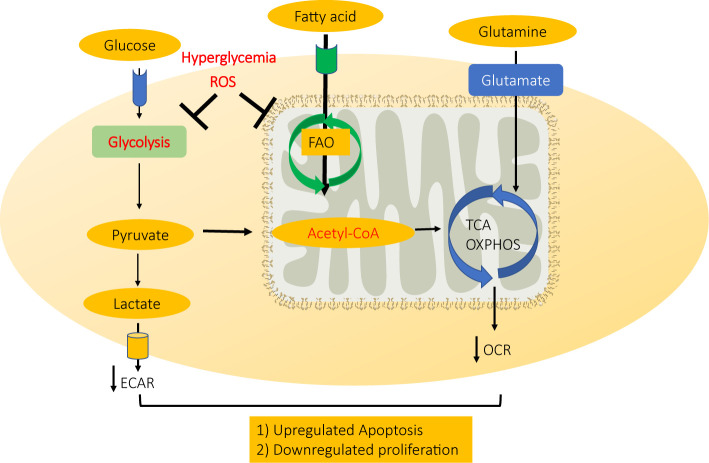
A schematic model for mechanism CMEC dysfunction in diabetes. High glucose-induced ROS accumulation in diabetic cardiac microvascular endothelial cells disrupts normal ATP production by inhibiting both mitochondrial electron transport system and glycolysis, therefore inhibiting CMEC proliferation and inducing apoptosis.

In summary, high glucose-induced ROS accumulation in diabetic cardiac microvascular endothelial cells could disrupt normal ATP production by inhibiting both mitochondrial electron transport system and glycolysis, therefore inhibiting CMEC proliferation and inducing CMEC apoptosis in diabetic hearts (**Figure 6**).

## Data Availability Statement

The original contributions presented in the study are included in the article/supplementary material. Further inquiries can be directed to the corresponding author.

## Ethics Statement

The animal study was reviewed and approved by Animal treatment protocols were approved by, and conducted in accordance with, animal welfare regulations of the Institutional Animal Care and Use Committee of the Medical College of Georgia.

## Author Contributions

YT: conceptualization. HZ, YT, I-mK, and NW: writing and data interpretation. HZ, YS, and YT: experiments. All authors contributed to the article and approved the submitted version.

## Funding

This work was supported by the American Heart Association (AHA) Transformational Project Award 18TPA34170104 and the National Institutes of Health (NIH) R01HL146481 (to I-mK); NIH R01HL134354 (to NW and YT); as well as AHA Grant-in-Aid 16GRNT31430008 and NIH R01HL086555 (to YT).

## Conflict of Interest

The authors declare that the research was conducted in the absence of any commercial or financial relationships that could be construed as a potential conflict of interest.

## References

[1] Olefsky JM Garvey WT Henry RR Brillon D Matthaei S Freidenberg GR. Cellular Mechanisms of Insulin Resistance in Non-Insulin-Dependent (Type II) Diabetes. Am J Med (1988) 85:86–105. 10.1016/0002-9343(88)90401-9 3057897

[2] Haffner S Taegtmeyer H. Epidemic Obesity and the Metabolic Syndrome. Circulation (2003) 108:1541–5. 10.1161/01.CIR.0000088845.17586.EC 14517149

[3] Lee TC Barshes NR Agee EE O’Mahoney CA Brunicardi FC Goss JA. The Effect of Whole Organ Pancreas Transplantation and PIT on Diabetic Complications. Curr Diabetes Rep (2006) 6:323–7. 10.1007/s11892-006-0068-x 16879786

[4] Farr AM Sheehan JJ Brouillette M Smith DM Johnston SS Kalsekar I. Healthcare Costs Among Adults With Type 2 Diabetes Initiating DPP-4 Inhibitors. Adv Ther (2016) 33:68–81. 10.1007/s12325-015-0277-2 26724938 PMC4735222

[5] Farr AM Sheehan JJ Curkendall SM Smith DM Johnston SS Kalsekar I. Retrospective Analysis of Long-Term Adherence to and Persistence With DPP-4 Inhibitors in US Adults With Type 2 Diabetes Mellitus. Adv Ther (2014) 31:1287–305. 10.1007/s12325-014-0171-3 PMC4271133 25504156

[6] Ke LY Law SH Mishra VK Parveen F Chan HC Lu YH. Molecular and Cellular Mechanisms of Electronegative Lipoproteins in Cardiovascular Diseases. Biomedicines (2020) 8. 10.3390/biomedicines8120550 PMC7760527 33260304

[7] Nita M Grzybowski A. The Role of the Reactive Oxygen Species and Oxidative Stress in the Pathomechanism of the Age-Related Ocular Diseases and Other Pathologies of the Anterior and Posterior Eye Segments in Adults. Oxid Med Cell Longevity (2016) 2016:3164734. 10.1155/2016/3164734 PMC4736974 26881021

[8] Glass CE Singal PK Singla DK. Stem Cells in the Diabetic Infarcted Heart. Heart Fail Rev (2010) 15:581–8. 10.1007/s10741-010-9172-8 PMC3072799 20559720

[9] Rolo AP Palmeira CM. Diabetes and Mitochondrial Function: Role of Hyperglycemia and Oxidative Stress. Toxicol Appl Pharmacol (2006) 212:167–78. 10.1016/j.taap.2006.01.003 16490224

[10] Lapel M Weston P Strassheim D Karoor V Burns N Lyubchenko T. Glycolysis and Oxidative Phosphorylation are Essential for Purinergic Receptor-Mediated Angiogenic Responses in Vasa Vasorum Endothelial Cells. Am J Physiol Cell Physiol (2017) 312:C56–70. 10.1152/ajpcell.00250.2016 PMC5283894 27856430

[11] Culic O Gruwel ML Schrader J. Energy Turnover of Vascular Endothelial Cells. Am J Physiol (1997) 273:C205–13. 10.1152/ajpcell.1997.273.1.C205 9252458

[12] Haspula D Vallejos AK Moore TM Tomar N Dash RK Hoffmann BR. Influence of a Hyperglycemic Microenvironment on a Diabetic Versus Healthy Rat Vascular Endothelium Reveals Distinguishable Mechanistic and Phenotypic Responses. Front Physiol (2019) 10:558–8. 10.3389/fphys.2019.00558 PMC6524400 31133884

[13] Skov V Glintborg D Knudsen S Jensen T Kruse TA Tan Q. Reduced Expression of Nuclear-Encoded Genes Involved in Mitochondrial Oxidative Metabolism in Skeletal Muscle of Insulin-Resistant Women With Polycystic Ovary Syndrome. Diabetes (2007) 56:2349–55. 10.2337/db07-0275 17563058

[14] Takamura T. Hepatokine Selenoprotein P-Mediated Reductive Stress Causes Resistance to Intracellular Signal Transduction. Antioxid Redox Signaling (2020) 33:517–24. 10.1089/ars.2020.8087 PMC7409583 32295394

[15] Primer KR Psaltis PJ Tan JTM Bursill CA. The Role of High-Density Lipoproteins in Endothelial Cell Metabolism and Diabetes-Impaired Angiogenesis. Int J Mol Sci (2020) 21:3633. 10.3390/ijms21103633 PMC7279383 32455604

[16] Dodd MS Sousa Fialho MDL Montes Aparicio CN Kerr M Timm KN Griffin JL. Fatty Acids Prevent Hypoxia-Inducible Factor-1α Signaling Through Decreased Succinate in Diabetes. JACC Basic Transl Sci (2018) 3:485–98. 10.1016/j.jacbts.2018.04.005 PMC6115650 30175272

[17] Da Ros R Assaloni R Ceriello A. Antioxidant Therapy in Diabetic Complications: What is New? Curr Vasc Pharmacol (2004) 2:335–41. 10.2174/1570161043385538 15320813

[18] Jin Y Shen Y Su X Cai J Liu Y Weintraub NL. The Small GTPases Rab27b Regulates Mitochondrial Fatty Acid Oxidative Metabolism of Cardiac Mesenchymal Stem Cells. Front Cell Dev Biol (2020) 8:209–9. 10.3389/fcell.2020.00209 PMC7174509 32351955

[19] Su X Jin Y Shen Y Kim I-M Weintraub NL Tang Y. RNAase III-Type Enzyme Dicer Regulates Mitochondrial Fatty Acid Oxidative Metabolism in Cardiac Mesenchymal Stem Cells. Int J Mol Sci (2019) 20:5554. 10.3390/ijms20225554 PMC6888515 31703292

[20] Yeh P-T Huang H-W Yang C-M Yang W-S Yang C-H. Astaxanthin Inhibits Expression of Retinal Oxidative Stress and Inflammatory Mediators in Streptozotocin-Induced Diabetic Rats. PloS One (2016) 11:e0146438. 10.1371/journal.pone.0146438 26765843 PMC4713224

[21] Rosa SC Gonçalves J Judas F Mobasheri A Lopes C Mendes AF. Impaired Glucose Transporter-1 Degradation and Increased Glucose Transport and Oxidative Stress in Response to High Glucose in Chondrocytes From Osteoarthritic Versus Normal Human Cartilage. Arthritis Res Ther (2009) 11:R80–0. 10.1186/ar2713 PMC2714130 19490621

[22] Sato D Itami N Tasaki H Takeo S Kuwayama T Iwata H. Relationship Between Mitochondrial DNA Copy Number and SIRT1 Expression in Porcine Oocytes. PloS One (2014) 9:e94488–8. 10.1371/journal.pone.0094488 PMC3991605 24747689

[23] Veys K Fan Z Ghobrial M Bouché A García-Caballero M Vriens K. Role of the GLUT1 Glucose Transporter in Postnatal CNS Angiogenesis and Blood-Brain Barrier Integrity. Circ Res (2020) 127:466–82. 10.1161/CIRCRESAHA.119.316463 PMC7386868 32404031

[24] Yan D Cai Y Luo J Liu J Li X Ying F. FOXO1 Contributes to Diabetic Cardiomyopathy Via Inducing Imbalanced Oxidative Metabolism in Type 1 Diabetes. J Cell Mol Med (2020) 24:7850–61. 10.1111/jcmm.15418 PMC7348139 32450616

[25] Stanley WC Lopaschuk GD McCormack JG. Regulation of Energy Substrate Metabolism in the Diabetic Heart. Cardiovasc Res (1997) 34:25–33. 10.1016/S0008-6363(97)00047-3 9217869

[26] Liu H Yu S Zhang H Xu J. Angiogenesis Impairment in Diabetes: Role of Methylglyoxal-Induced Receptor for Advanced Glycation Endproducts, Autophagy and Vascular Endothelial Growth Factor Receptor 2. PloS One (2012) 7:e46720. 10.1371/journal.pone.0046720 PMC3463541 23056421

[27] Tofte N Suvitaival T Trost K Mattila IM Theilade S Winther SA. Metabolomic Assessment Reveals Alteration in Polyols and Branched Chain Amino Acids Associated With Present and Future Renal Impairment in a Discovery Cohort of 637 Persons With Type 1 Diabetes. Front Endocrinol (2019) 10:818–8. 10.3389/fendo.2019.00818 PMC6883958 31824430

[28] Shao C Lin S Liu S Jin P Lu W Li N. Hif1α-Induced Glycolysis in Macrophage is Essential for the Protective Effect of Ouabain During Endotoxemia. Oxid Med Cell Longevity (2019) 2019:7136585. 10.1155/2019/7136585 PMC6512009 31182997

[29] Morciano G Patergnani S Bonora M Pedriali G Tarocco A Bouhamida E. Mitophagy in Cardiovascular Diseases. J Clin Med (2020) 9:892. 10.3390/jcm9030892 PMC7141512 32214047

[30] Park MH Han JS. Protective Effect of Padina Arborescens Extract Against High Glucose-Induced Oxidative Damage in Human Umbilical Vein Endothelial Cells. Prev Nutr Food Sci (2013) 18:11–7. 10.3746/pnf.2013.18.1.011 PMC3867148 24471104

[31] Reusch JE. Diabetes, Microvascular Complications, and Cardiovascular Complications: What is It About Glucose? J Clin Invest (2003) 112:986–8. 10.1172/JCI200319902 PMC198532 14523035

[32] Redina OE Smolenskaya SE Klimov LO Markel AL. Candidate Genes in Quantitative Trait Loci Associated With Absolute and Relative Kidney Weight in Rats With Inherited Stress Induced Arterial Hypertension. BMC Genet (2015) 16 Suppl 1:S1–1. 10.1186/1471-2156-16-S1-S1 PMC4331803 25707311

[33] Poungvarin N Lee JK Yechoor VK Li MV Assavapokee T Suksaranjit P. Carbohydrate Response Element-Binding Protein (ChREBP) Plays a Pivotal Role in Beta Cell Glucotoxicity. Diabetologia (2012) 55:1783–96. 10.1007/s00125-012-2506-4 PMC4010252 22382520

[34] Li Q Kim Y-R Vikram A Kumar S Kassan M Gabani M. P66shc-Induced MicroRNA-34a Causes Diabetic Endothelial Dysfunction by Downregulating Sirtuin1. Atheroscler Thrombosis Vasc Biol (2016) 36:2394–403. 10.1161/ATVBAHA.116.308321 PMC5293179 27789474

[35] Chen X-F Wang L Wu Y-Z Song S-Y Min H-Y Yang Y. Effect of Puerarin in Promoting Fatty Acid Oxidation by Increasing Mitochondrial Oxidative Capacity and Biogenesis in Skeletal Muscle in Diabetic Rats. Nutr Diabetes (2018) 8:1–1. 10.1038/s41387-017-0009-6 29330446 PMC5851431

[36] Ljubkovic M Gressette M Bulat C Cavar M Bakovic D Fabijanic D. Disturbed Fatty Acid Oxidation, Endoplasmic Reticulum Stress, and Apoptosis in Left Ventricle of Patients With Type 2 Diabetes. Diabetes (2019) 68:1924–33. 10.2337/db19-0423 31391173

[37] Jung UJ Cho Y-Y Choi M-S. Apigenin Ameliorates Dyslipidemia, Hepatic Steatosis and Insulin Resistance by Modulating Metabolic and Transcriptional Profiles in the Liver of High-Fat Diet-Induced Obese Mice. Nutrients (2016) 8:305. 10.3390/nu8050305 PMC4882717 27213439

